# Primary results from SPRINT MIND 2020

**DOI:** 10.1002/alz.095413

**Published:** 2025-01-09

**Authors:** Jeff D. Williamson

**Affiliations:** ^1^ Wake Forest University School of Medicine, Winston‐Salem, NC USA

## Abstract

**Background:**

The Systolic Blood Pressure Intervention (SPRINT) randomized clinical trial evaluated the effect of intensive blood pressure (BP) control, defined by targeting systolic BP < 120 mm Hg, versus the standard target of < 140 mm Hg. Participants were recruited from 102 sites in the United States and Puerto Rico, aged 50 years or older with hypertension but without diabetes or history of stroke. SPRINT stopped early, after a median treatment of only 3.34 years of intervention, due to benefit on its primary outcome (a composite of cardiovascular events) and all‐cause mortality. SPRINT‐MIND, a sub‐study of SPRINT that evaluated cognitive outcomes, found intensive BP control reduced risk of mild cognitive impairment (MCI) by 19% and reduced the risk of developing either MCI or probable dementia (PD) by 15%. There was also a statistically non‐significant reduction of 17% in PD alone. We investigated the long‐term impact of intensive BP control on MCI and PD by conducting extended cognitive assessment for SPRINT‐MIND, adding follow‐up for cognitive outcomes through December 31, 2023.

**Methods:**

The extended follow‐up was designed to administer a cognitive assessment battery by phone to 7221 participants in the main trial that were not withdrawn or had been adjudicated as having probable dementia and consented to future research. Participants were contacted by phone were asked to provide verbal informed consent. Phone calls began on December 2^nd^ 2019.

**Results:**

There were 9361 participants originally randomized (mean age, 67.9 years; 3332 women [35.6%]) and of these, 7221 (77%) were free of dementia, and consented to long‐term follow‐up cognitive assessment. During this extended total median follow‐up of 6.9 years, adjudicated PD occurred in a total of 541 participants and MCI occurred in a total of 810 participants.

**Conclusion:**

The results from SPRINT MIND 2020 will demonstrate the long‐term benefit of intensive systolic blood pressure control on cognitive function.

